# Single tooth restoration in the maxillary esthetic zone using a one-piece ceramic implant with 1 year of follow-up: case series

**DOI:** 10.1186/s40729-021-00308-z

**Published:** 2021-04-06

**Authors:** Miren Vilor-Fernández, Ana-María García-De-La-Fuente, Xabier Marichalar-Mendia, Ruth Estefanía-Fresco, Luis-Antonio Aguirre-Zorzano

**Affiliations:** 1 Department of Stomatology II, University of the Basque Country (UPV/EHU), Barrio Sarriena s/n, 48940 Leioa, Bizkaia Spain; 2 Department of Nursing I, University of the Basque Country (UPV/EHU), Barrio Sarriena s/n, 48940 Leioa, Bizkaia Spain

**Keywords:** Zirconia implant, Ceramic implant, One-piece, Single tooth, Marginal bone loss, Survival rate

## Abstract

**Background:**

Oral implants have helped clinicians to improve the quality of life for many patients. The material of choice for dental implants currently remains titanium type IV, whose mechanical and biological properties have been proven throughout the history of implantology. Yet, this material is not exempt from complications. For these reasons, ceramic alternatives to titanium have emerged. Thus, the purpose of this study is to evaluate peri-implant hard and soft tissue stability with the use of a one-piece ceramic implant (Straumann® PURE Ceramic Implant) during 1 year of follow-up.

**Study design:**

One-piece all-ceramic zirconia (*ZrO*_2_) implants were placed to replace single missing teeth in the esthetic zone. Six to 8 weeks after the procedure, the definitive prosthesis was fabricated. At the time of prosthesis, placement (*T*_0_) photographs and periapical radiographs were taken, and the following clinical parameters were recorded: probing depth (PD), plaque index (PI), bleeding on probing (BOP), suppuration on probing (SOP), distance from gingival margin to incisal edge (GM-IE), and Jemt papilla index (JPI). Follow-up appointments were scheduled at 4 (*T*_4_), 8 (*T*_8_), and 12 (*T*_12_) months, when the same parameters were recorded. In addition, plaque control was reinforced and prophylaxis was carried out. In this last appointment, a final periapical radiograph was taken to assess marginal bone loss.

**Results:**

A total of 32 zirconia implants were placed in 28 patients (16 women and 12 men, aged between 34 and 67 years). The survival and success rate were 93.75%. The increase in probing depth from baseline to 12 months was 0.78 mm. Assessments of plaque index and bleeding on probing showed a slight increase throughout the study.

**Conclusions:**

The results obtained with the Straumann® PURE Ceramic implants show them to exhibit very good clinical behavior. The survival rate of the implants of our pilot study was 93.75%. For these reasons, we can say that zirconia implants could be an alternative to titanium implants in the esthetic zone.

## Background

Oral implants have helped clinicians to improve the quality of life for many patients. The material of choice for dental implants currently remains titanium type IV, whose mechanical and biological properties have been proven throughout the history of implantology [1, 2]. Yet, this material is not exempt from complications. First, this type of metallic implant can have esthetic limitations, especially when used in anterior regions and in patients with a thin biotype. The complications to which we refer are the appearance of a metallic margin due to recession, or grayish colorations due to the translucency of the metal through the peri-implant mucosa [3–5]. Second, some studies have reported immunological reactions to titanium particles which lead to biological complications [6, 7], while others have demonstrated allergic reactions to titanium, including Sicilia et al. (2008) who observed a prevalence of 0.6% [8, 9]. Finally, it must also be taken into account that the number of patients who demand “metal-free” implants is increasing.

For these reasons, ceramic alternatives to titanium have emerged. The first ceramic implants arrived on the market more than 40 years ago. They were made of alumina, a material prone to fracture when loaded unfavorably [10], and so, they are no longer available. Currently, the material of choice for the manufacture of ceramic implants is yttria tetragonal zirconia polycrystal (Y-TZP), characterized by its high resistance to fracture, a low modulus of elasticity, a low affinity to plaque and high biocompatibility [11–14].

The objective of this pilot study was to evaluate the stability of hard and soft peri-implant tissues with the use of a one-piece ceramic implant (Straumann® PURE Ceramic Implant Monotype) with a 1-year follow-up.

## Materials and methods

### Study design

This project began, initially, as a pilot study, which consists of a retrospective part and a prospective follow-up part. This observational ambispective clinical study was approved by the Clinical Research Ethics Committee of the Basque Country (CEIC-E) (Internal Code PI2016088, 07/2016). In addition, it is registered at www.ClinicalTrials.com (n° NCT03352284).

### Study population

Patients who met the following inclusion criteria were included in the study:

1. Patients treated in Own Master of Periodontology and Osseointegration in the University of the Basque Country (UPV/EHU)
2. Age > 18 years
3. One single tooth missing in the anterior maxilla (1.5–2.5)
4. Plaque index <20% [15]
5. Bleeding on probing index <20% [16]
6. Periodontally healthy or treated periodontal conditions
7. Is able to fully understand the nature of the proposed surgery and is able to provide signed informed consent

Conversely, patients who presented any of the following exclusion criteria were not included in the study:

1. General contraindications to dental and/or surgical treatment
2. Is taking medications or receiving treatments which have an effect on healing in general (e.g., steroids or large doses of anti-inflammatory drugs)
3. Untreated periodontal conditions
4. Not willing to participate

In this way, a total of 28 patients (16 women and 12 men) with a mean age of 54.1 years [34-67 years] who required dental implants to replace a single tooth in the maxilla were recruited to participate in the study, and all of them were properly informed and signed a written informed consent.

### Clinical and radiographic evaluation

All-ceramic one-piece implants (PURE Ceramic Implants Monotype, Institut Straumann AG, Basel, Switzerland) were used.

Before surgery, a clinical and radiographic diagnostic assessment was carried out to choose the appropriate implant for each case. To locate the 3D implant position, the following minimal distances were taken into consideration: minimum 1.5 to 2 mm from the natural adjacent teeth, 1 mm palatal to the ideal point of emergence, and 2 mm apical to the midfacial gingival margin of the final implant prosthesis [17]. A minimum of 1.5 mm to 2 mm thickness of buccal bone was preserved as well. The implant diameter and the length were chosen according to each individual case. These implants had two different abutment heights: 4 and 5.5 mm, and for the selection of the implant, all these considerations (position of natural teeth, width of alveolar ridge and occlusion) were taken into consideration in the planification of the treatment of each patient. Finally, study models were made to manufacture splints for use as surgical guides for each patient.

**Fig. 1:**
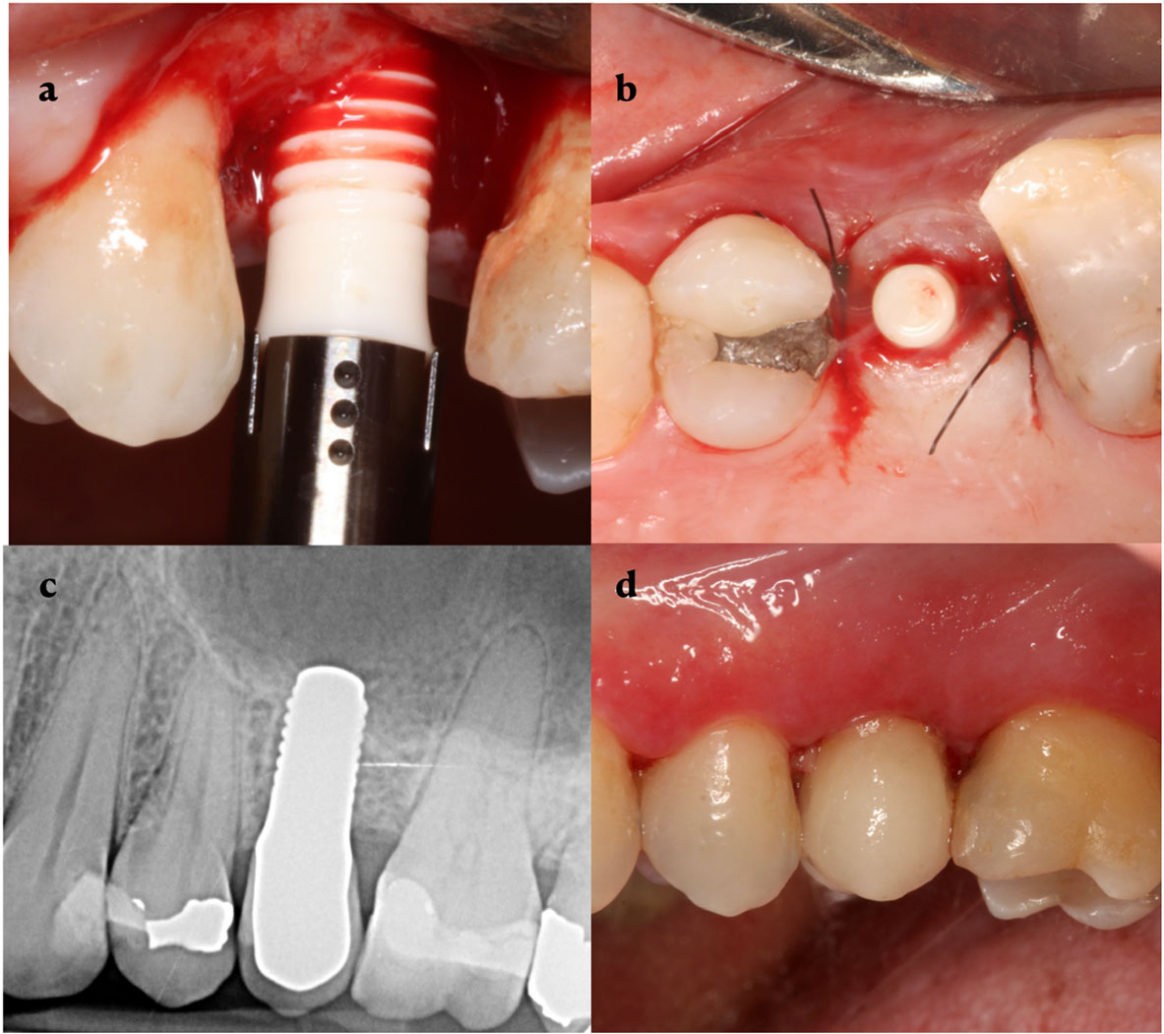
Clinical case. **a** Surgery, **b** implant placed, **c** radiographic evaluation at 12 months, and **d** prosthesis at 12 months

All surgeries were performed under local anesthesia with articaine (Meganest ® 1:200.000, Clarben, Madrid, Spain). The surgical technique consisted in the elevation of a full-thickness mucoperiosteal flap, both vestibular and palatal, through a mid-crestal incision. After the alveolar bone had been exposed, the drilling sequence was carried out according to the manufacturer’s instructions before the placement of the fixture. In some cases where bone volume was insufficient, dehiscence or fenestrations were treated by the guided bone regeneration technique with xenograft (Bio-Oss®, Geistlich Pharma AG, Wolhusen, Switzerland) and resorbable collagen membrane (Collagene AT®, Centro de Odontoiatria Operativa S.R.L, Podova, Italy). Once the implant had been inserted, a healing cap was placed in almost all cases and the flap was closed and sutured (Fig. 1a–d).

In 18 of the cases (56.3%), an immediate provisional crown was made due to the esthetic demands of the patients. In this case, primary stability with an insertion torque > 30 Ncm was confirmed and a pre-formed polycarbonate crown of the appropriate size was chosen. That crown was overlaid with a self-curing resin (Tab 2000, Kerr, Scafati, Italy) and composite (TPH Spectrum®, Dentsply Sirona, York, Pensilvania, USA) and then polished perfectly. The crown was cemented with Temp-Bond (Temp-Bond™, Kerr, Scafati, Italy), and the occlusion was verified to check that there was no contact during centric or eccentric movements to prevent loading during osseointegration.

Post-operative instructions included amoxicillin 750 mg every 8 h for 8 days, dexketoprofen 25 mg every 8 h for 4 days, and rinsing with chlorhexidine digluconate 0.12% twice a day for 15 days. Sutures were removed after 7 days.

After 6 to 8 weeks of healing and following the manufacturer’s recommendations, final impressions were taken using the corresponding impression cap with a closed tray and VPS impression material of putty (Empress™ 2 Putty Soft, 3M ESPE, Seefeld, Germany) and ultra-light viscosity (Empress™ 2 Ultra-Light Body Quick, 3M ESPE, Seefeld, Germany). Due to the one-piece design of the ceramic implant, all-ceramic zirconia crowns were inserted in all cases. Permanent glass ionomer cement (Ketac Cem™, 3M ESPE, Seefeld, Germany) was used to cement the crowns directly onto the implant abutment. Special attention was paid to remove all remaining cement.

After placement of the definitive prosthesis (T_0_), we carried out photography, intra-oral radiography, and measurements of probing depth, bleeding on probing, maximum distance from the gingival margin to the incisal edge, and Jemt papilla index, and took these parameters as the baseline from which any changes were evaluated.

In each of the follow-up appointments, 4 (T_4_), 8 (T_8_) and 12 months (T_12_) after the placement of the prosthesis, plaque control was reinforced, whatever supragingival plaque that could be removed was removed, a photograph of the restoration was taken, and the following parameters were recorded by one of the researchers (MV):

- Probing depth (PD): pocket probing on dental implants was recorded with light force (approximately 0.25N) at six points around the implant.
- Bleeding on probing (BOP) (at six points around the implant) [16]
- Suppuration on probing (SOP): local SOP score was recorded as the percentage of total surfaces (six points per implant) that exhibited suppurating on gentle probing with a light force (approximately 0.25N)
- Plaque index [15]
- Peri-implant recession (Pi-Rec): the difference between maximum distance from the gingival margin to the incisal edge measured at baseline and 1 year-follow-up, measured on the mid buccal site.
- Jemt papilla index (JPI) [18]

At the last follow-up appointment, an X-ray was also performed to assess bone changes, namely any loss or gain as measured at the mesial and distal aspect of each implant relative to the baseline measurements at the beginning of the study. The final photograph was used to analyze soft tissue changes, namely peri-implant recession (Pi-Rec) and the state of the papilla according to the Jemt papilla index [18].

All radiographic measurements were taken by the same investigator (RE), at baseline (T_0_) and at 12 months (T_12_). Changes in the bone level were measured both mesially and distally of the implant on a periapical X-ray taken with a standardized film holder (Rinn® Flip-Ray Film Holder, Rinn, Dentsply International Inc. Elgin, IL, EEUU). The length of the polished neck of the implant (1.8 mm) served as a reference to calibrate the X-ray before measuring marginal bone loss (MBL) from the neck of the implant to the first bone-implant contact (BIC). A positive value indicated that the first BIC was located above the first thread of the implant, and a negative one indicated that it was located below.

### Statistical analysis

The data were analyzed by XM using IBM SPSS software version 22. For descriptive statistics, we used the mean, standard deviation, rank, and percentages. For analytical statistics, the Wilcoxon signed-rank test for related samples was carried out. *P* values of less than 0.05 were considered statistically significant.

## Results

Thirty-two Straumann® PURE Ceramic implants were placed by only one surgeon (MV) in 28 patients. The diameters of the implants were 3.3 and 4.1 mm, and the lengths ranged from 8 to 14 mm. These implants had two different abutment heights: 4 and 5.5 mm. Twenty-three implants were placed in the premolar region (71.9%), four in the canine position (12.5%), and five in the lateral incisor position (15.6%). The patient demographics and implant characteristics are described in Table 1.

**Table 1:** Summary of patient demographics and implant characteristics (*n* = 32 implants)

	*N* (%)
**Patient data**	
Sex, *n*	16 females
	12 males
Gender: *n* and (%) female	16 (57.15)
Mean age (years)	54.1
Age range (years)	34–67
Smokers	8 (31.3)
**Implant data**	
Site of implant placement (*n* and %)	
Lateral incisor	5 (15.6)
Canine	4 (12.5)
1st Premolar	19 (59.4)
2nd Premolar	4 (12.5)
Length of implant, *n* (%)	
8 mm	2 (6.25)
10 mm	20 (62.5)
12 mm	8 (25)
14 mm	2 (6.25)
Immediate implant	4 (12.5)
Bone augmentation, *n* (%)	9 (28.12)
Soft tissue augmentation, *n* (%)	4 (12.5)
Provisional restoration	18 (56.3)
Follow-up (months)	12

In nine of the surgeries (28.12%), an additional bone regenerative procedure was required due to dehiscences that arose from implant placement. Additionally, in four cases (12.5%), a connective tissue graft was placed to increase the soft tissue volume. It should be noted that no patient had postoperative complications, instead of two cases (6.25%) that, during the healing phase, showed mobility and the implant had to be removed. Thus, the survival rate obtained in this study was 93.75%.

### Soft tissues

**Fig. 2:**
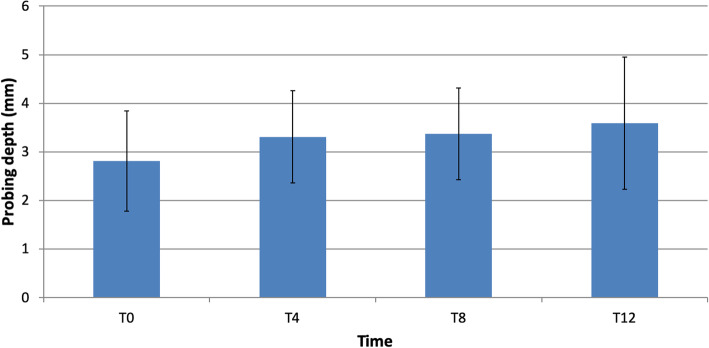
Changes in probing depth

Probing depth increased slightly over the study time. Initially, at the time of the final crown placement (T0), the mean PD was 2.81 ± 1.03 mm and it progressively increased in T4, T8, and T12 with the mean PD of 3.31 ± 0.95 mm, 3.37 ± 0.94 mm, and 3.59 ± 1.37 mm, respectively. We observed a statistically significant difference in PD between baseline and 12 months of 0.78 mm *(p = 0.002)* (Fig. 2).

**Fig. 3:**
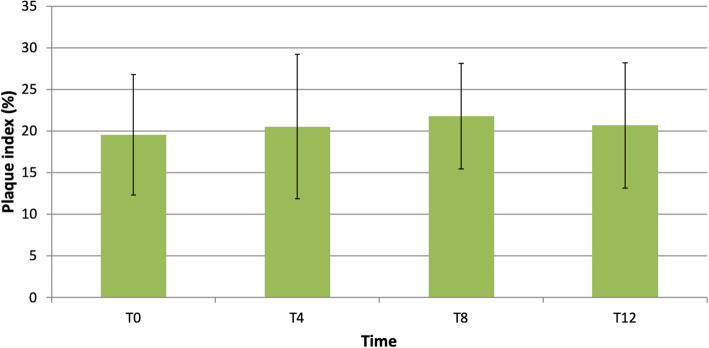
Changes in plaque index

In relation to the PI [15] at the beginning, at 4, 8, and 12 months after the placement of the prosthesis, this revealed a percentage of surfaces with dental plaque of 19.55 ± 7.22%, 20.53 ± 8.67%, 21.77 ± 6.35%, and 20.67 ± 7.53%, respectively (Fig. 3).

**Fig. 4:**
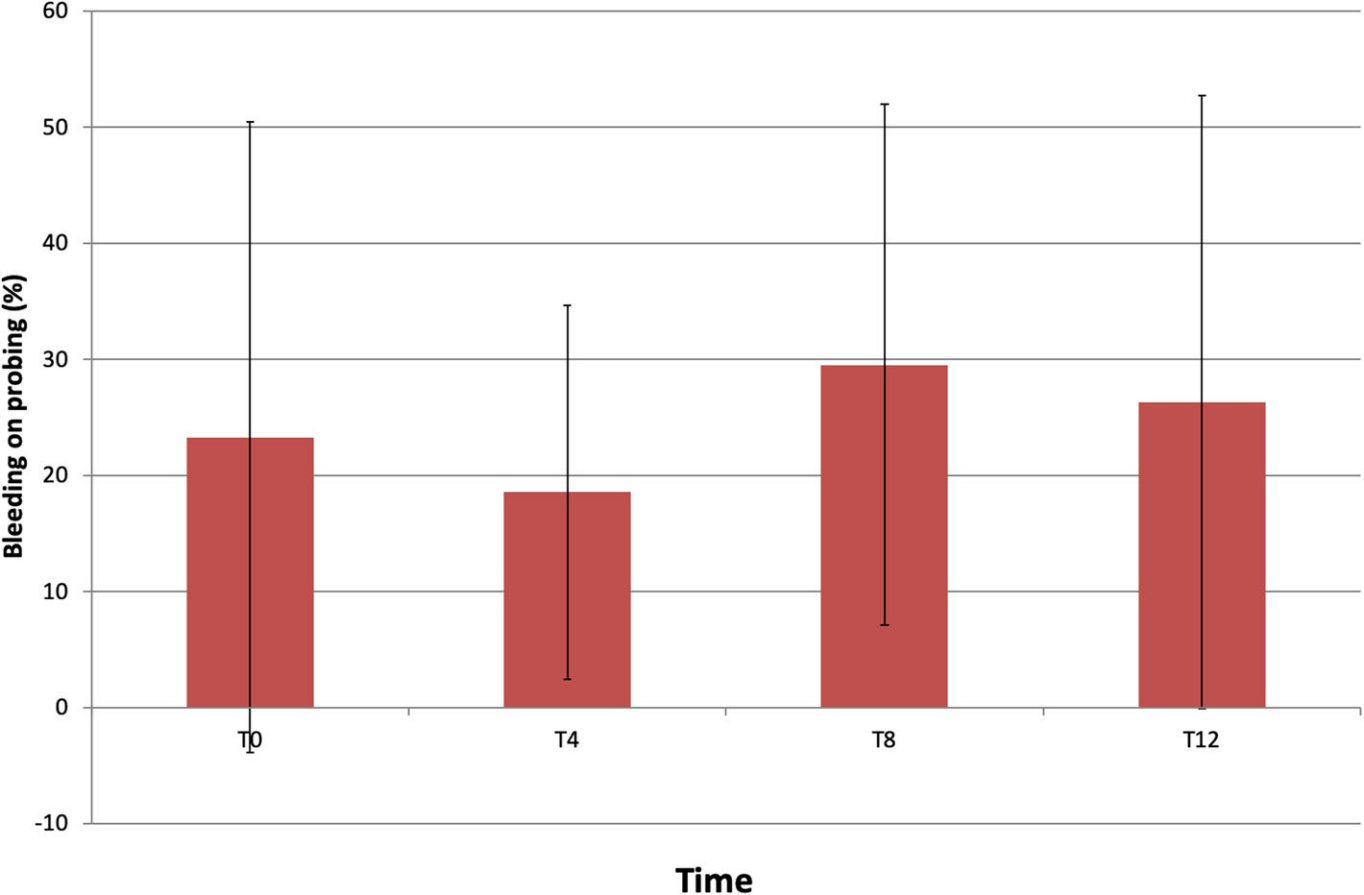
Changes in bleeding on probing index

Bleeding on probing (BOP) remained stable throughout the study; thus, in T0, T4, T8, and T12 were 23.26 ± 27.18%, 18.53 ± 16.11%, 29.5 ± 22.43%, and 26.3 ± 26.41%, respectively (Fig. 4). There were no significant differences between any of the study times.

It should be noted that of the 30 implants studied, after 1 year, 28 of them did not have any Pi-Rec. Only two implants experienced a Pi-Rec of 1 mm, assuming an average Pi-Rec of 0.07 mm after 1 year of follow-up. None of both implants showing recession were either an immediate implant or treated with regenerative procedures.

On the other hand, the Jemt papillae index (JPI) showed an improvement regarding the filling of the interproximal spaces, probably, due to the maturation of the papillae. Thus, initially 37.5% of the cases showed an JPI = 0, 37.5% JPI = 1, 12.5% JPI = 2, and 9.5% JPI = 3. After 12 months, the percentages were 9.4%, 40.6%, 31.3%, and 12.5%, respectively. The mean JPI of T0 was 0.94, while in T12, it was 1.5, indicating that in several of the cases the papilla was in a more coronal position at the end of the study.

### Hard tissues

**Fig. 5:**
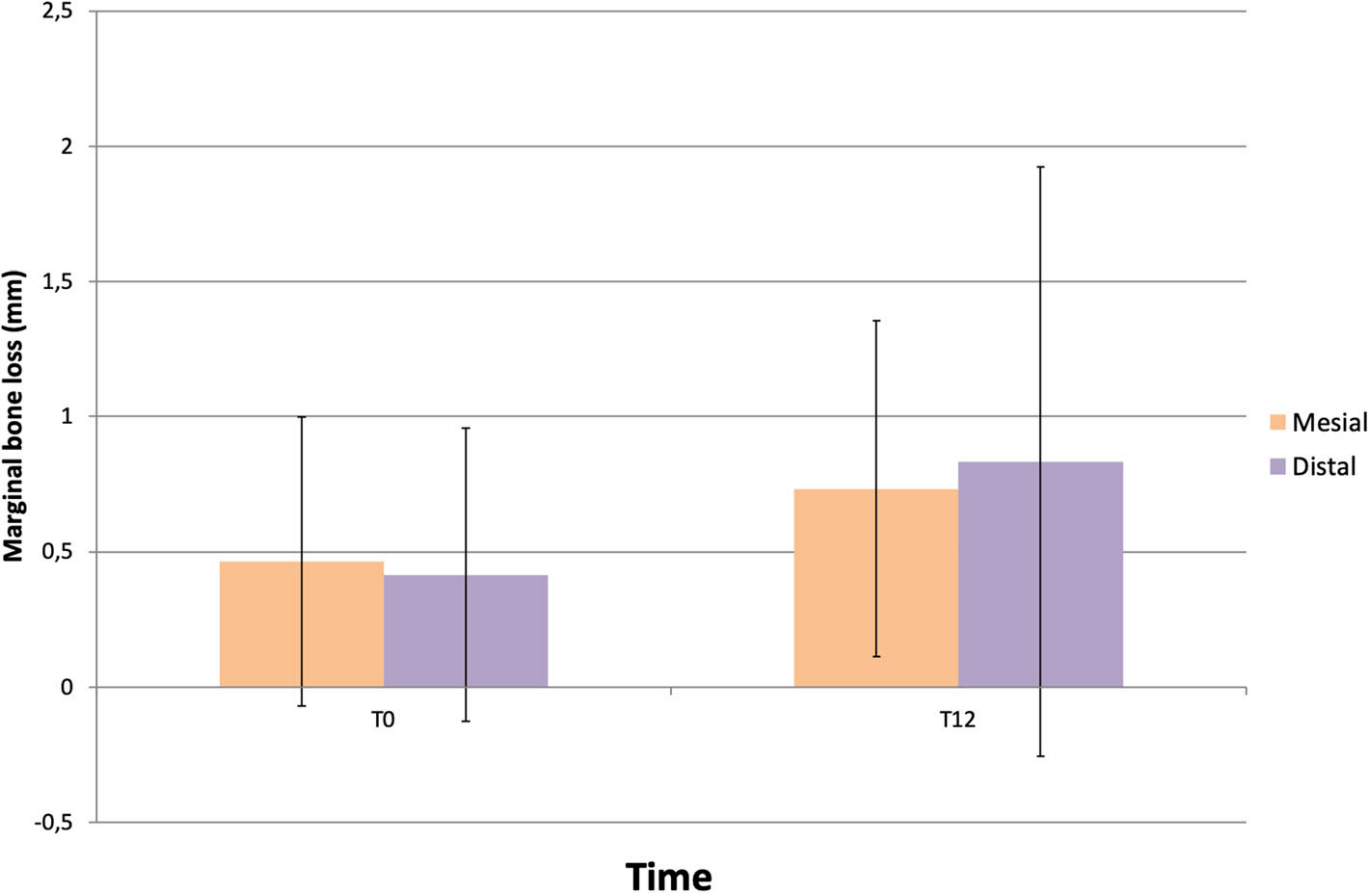
Marginal bone loss at mesial and distal sites

Marginal bone loss (MBL) from implant placement to prosthetic loading on the mesial aspect was 0.46 ± 0.53 mm (*p* <0.001) and on the distal aspect 0.41 ± 0.54 mm (*p* < 0.001). Twelve months after the prosthetic loading, the mean MBL compared to surgery was 0.73 ± 0.62 mm mesially and 0.83 ± 1.09 mm distally, both statistically significant (*p* < 0.001) (Fig. 5).

## Discussion

Our pilot study evaluated the stability of hard and soft tissues around Straumann® PURE Ceramic implants, and this stability is considered a determining factor for a successful result. According to the conclusions established during the first European Workshop on Periodontology, MBL <1.5 mm after the first year of loading and an additional 0.2 mm annually should be taken as a successful treatment [19]. To compare the results available in the literature, we selected the studies that include single crowns cemented over one-piece ceramic implants. The success rate is a variable which few studies have mentioned, but the survival rate is reported by numerous articles. A systematic review that included nine articles with 326 patients and 398 implants, and a follow-up time which ranged from 12 months to 5 years reported a survival rate of 95.6% [20], the same as the 95% reported by Payer et al. [21] and similar to the 92% reported by Hashim et al. [22], both lower than the 100% survival rate reported here and by Borgonovo et al. [23].

The main purpose of this study was to measure marginal bone level changes after 1 year of follow-up. The results of our study should be interpreted with caution since the bone loss observed between surgery and T0 should be considered rather than MBL, bone remodeling, because areas of the polished neck were subcrestal at the time of implant placement. Twelve months after surgery, MBL was 0.73 mm at the mesial aspect of the implant and 0.83 mm at the distal aspect. These results are comparable to those of*.* Grassi et al. [24], who observed an average MBL of 0.83 mm after 12 months. A recent systematic review comparing this kind of implants versus titanium implants (Ti implants) reported an MBL of 0.89 ± 0.18 mm favoring zirconia implants (Zi implants) after 12–24 months [25]. The difference (0.14 mm) with the Ti implants was statistically significant (*p*=0.053). Another study published by Kohal et al. [26] showed a higher MBL (1.31 ± 1.49 mm) between the time of implant placement and 1 year of follow-up. Conversely, there are other studies reporting higher MBL, such as that of Gahlert et al. [27], who observed an MBL of 1.02 mm after 1 year in use.

Our study did not exclude cases that required minor bone augmentation procedures during implant surgery, and the prosthesis procedure began at 6–8 weeks post-surgery, independently of requiring GBR or not because it was considered that the minor GBR needed for treat small dehiscences would not interfere with osteointegration [28]. Like other studies, we used bovine bone graft covered with a resorbable collagen membrane [29–31]. A review that unified the data of these articles did not find statistically significant differences in MBL between applying guided bone regeneration (0.79 mm) and not applying it (0.97 mm) [22]. In the present investigation, we corroborate this statement since the MBL after 1 year of loading was 0.61 mm in the cases where GBR was not carried out compared to 0.94 mm in the cases where regenerative procedures were applied *(p=0.342).* Although one-piece implants do not allow primary closure of the wound, procedures of minor bone augmentation did not appear to have any significant effect on MBL [22, 28].

One of the advantages of one-piece implants is that they might minimize bone loss by avoiding micromovements and implant-abutment microgaps [32]. A systematic review comparing one-piece implants versus two-piece implants observed a statistically significant difference in terms of MBL (0.93 ± 0.19 mm vs 1.46 ± 0.57 mm) [25]. Conversely, the difficulty posed by these one-piece implants is that they limit the prosthetic options for carrying out the rehabilitation. Even preparing the implant head is discouraged since, as observed by Silva et al. [14], it has a negative influence on implant fracture. Furthermore, the aging of this material in the presence of humidity at room temperature is widely known. However, in vitro studies [33, 34] evaluating the impact of low temperature degradation of *Zr*0_2_ observe that the effect of aging was minimal for these ceramic implants, suggesting a reliable clinical application of this material. For this reason, it is an area inconstant research and development to achieve implants with greater resistance to fatigue and degradation.

The evaluation of clinical soft tissue parameters indicated an absence of peri-implant biological complications, showing an average PD of 3.59 mm and BOP of 26.3% after 12 months. These data are comparable to those obtained in a recent study published by Balmer et al. [35] where they recorded an average PD of 3.47 ± 0.67 mm, which was slightly lower than what we observed, and BOP of 57.5 ± 32.9%, which, conversely, was higher than what we obtained. In some cases, BOP could be identified as a clinical sign of mucositis due to incorrect plaque control or microgaps between the crown and the implant [36]. Our probing depth was slightly higher than that observed by Cionca et al. [37] in a systematic review, where it ranged from 1.8 to 3.2 mm.

## Conclusions

The results obtained with the Straumann® PURE Ceramic implants show them to exhibit very good clinical behavior in terms of hard and soft tissue stability. The survival rate of the implants of our pilot study was 93.75%. For these reasons, we can conclude that implants could be an alternative to titanium implants in the esthetic zone. However, more long-term clinical studies are necessary to confirm the clinical efficacy and the mechanical resistance of *Zr*0_2_ as a material for the manufacture of dental implants.

## Data Availability

The datasets used and/or analyzed during the current study are available from the corresponding author on reasonable request.

## Abbreviations

*Zr*0_2_: Zirconia
Ti: Titanium
PD: Probing depth
PI: Plaque index
BOP: Bleeding on probing
SOP: Suppuration on probing
GM-IE: Distance from gingival margin to incisal edge
JPI: Jemt papilla index
Pi-Rec: Periimplant recession
MBL: Marginal bone loss
