# Publisher Correction to: Single tooth restoration in the maxillary esthetic zone using a one-piece ceramic implant with 1 year of follow-up: case series

**DOI:** 10.1186/s40729-021-00395-y

**Published:** 2021-11-24

**Authors:** Miren Vilor-Fernández, Ana-María García-De-La-Fuente, Xabier Marichalar-Mendia, Ruth Estefanía-Fresco, Luis-Antonio Aguirre-Zorzano

**Affiliations:** 1 Department of Stomatology II, University of the Basque Country (UPV/EHU), Barrio Sarriena s/n, 48940 Leioa, Bizkaia Spain; 2 Department of Nursing I, University of the Basque Country (UPV/EHU), Barrio Sarriena s/n, 48940 Leioa, Bizkaia Spain

## Publisher Correction to: Int J Implant Dent (2021) 7:26 https://doi.org/10.1186/s40729-021-00308-z

During the publication process of the original article [1] an error was made in the article alongside a pre-existing mathematical error.

As shown in the Result section: the number of lost implants was 2, which results in a rate of 6.25%, as indicated in the article itself.

However, the survival rate does not correspond to the real survival rate. Instead of a survival rate of 96.9%, it should appear a survival rate of 93.75%.

In the conclusion section, instead of “**The survival rate of the implants of our pilot study was 9.9%.**”, it should read: “**The survival rate of the implants of our pilot study was 93.75%.**”. This error was introduced during the publication process.

These errors were present in three locations:

1. In the **Results** and **Conclusion** subsection of the Abstract.
2. In the **Result** section of the article.
3. In the **Conclusion** section of the article.

The original article has been updated as this does not affect the conclusions. The publisher apologizes for the inconvenience caused to the authors and readers.

## References

[1] Vilor-Fernández M, García-De-La-Fuente AM, Marichalar-Mendia X (2021). Single tooth restoration in the maxillary esthetic zone using a one-piece ceramic implant with 1 year of follow-up: case series. Int J Implant Dent.

